# A cost analysis to address issues of budget constraints on the implementation of the indoor residual spray programme in two districts of Maputo Province, Mozambique

**DOI:** 10.1186/s12936-020-03556-3

**Published:** 2021-01-06

**Authors:** Neide Canana

**Affiliations:** National Institute of Health, 3943, 1 National St., Marracuene Village, Maputo Province Mozambique

**Keywords:** Cost analysis, Indoor residual spray, Intervention operationalization, Government budget, Official development assistance, Budget restriction, Mozambique

## Abstract

**Background:**

It is frequently said that funding is essential to ensure optimal results from a malaria intervention control. However, in recent years, the capacity of the government of Mozambique to sustain the operational cost of indoor residual spraying (IRS) is facing numerous challenges due to restrictions of the Official Development Assistance. The purpose of the study was to estimate the cost of IRS operationalization in two districts of Maputo Province (Matutuíne and Namaacha) in Mozambique. The evidence produced in this study intends to provide decision-makers with insight into where they need to pay close attention in future planning in order to operationalize IRS with the existent budget in the actual context of budget restrictions.

**Methods:**

Cost information was collected retrospectively from the provider perspective, and both economic and financial costs were calculated. A “one-way” deterministic sensitivity analysis was performed.

**Results:**

The average economic costs totaled US$117,351.34, with an average economic cost per household sprayed of US$16.35, and an average economic cost per person protected of US$4.09. The average financial cost totaled US$69,174.83, with an average financial cost per household sprayed and per person protected of US$9.84 and US$2.46, respectively. Vehicle, salary, and insecticide costs were the greatest contributors to overall cost in the economic and financial analysis, corresponding to 52%, 17%, and 13% in the economic analysis and 21%, 27%, and 22% in the financial analysis, respectively. The sensitivity analysis was adapted to a range of ± (above and under) 25% change. There was an approximate change of 14% in the average economic cost when vehicle costs were decreased by 25%. In the financial analysis, the average financial cost was lowered by 7% when salary costs were decreased by 25%.

**Conclusions:**

Altogether, the current cost analysis provides an impetus for the consideration of targeted IRS operationalization within the available governmental budget, by using locally-available human resources as spray operators to decrease costs and having IRS rounds be correctly timed to coincide with the build-up of vector populations.

## Background

It is frequently said that funding is essential to ensure optimal results of a malaria intervention. Ensuring funding include calculating the total cost, who it is paid by, and putting in place mechanisms that can ensure sustainability. In Mozambique, malaria is endemic and the entire population, 29,365.27, is at risk of contracting the disease [1, 2]. The country was the fifth highest contributor of numbers of malaria cases in 2018 globally, which is 4% of all global cases according to the 2019 World Health Organization (WHO) report [3]. While the last decade observed significant reductions in the burden of malaria globally, according to the WHO, in Mozambique an increase in the number of malaria cases has been observed since 2015 [1, 2]. Data from the 2018 Malaria Indicator Survey by the National Institute of Health (INS) showed that in 2018 malaria accounted for over 10 million cases diagnosed in public health facilities and communities, resulting in an estimated 14,700 deaths, corresponding to 21% and 42% of the total death in all ages and in children under the age of five respectively, thereby placing the disease as one of the leading causes of morbidity and mortality and the first cause of mortality in children under 5 years of age in the country [4]. Even though Mozambique’s entire population is at risk of malaria, malaria prevalence varies across the country. Prevalence is higher in the northern and central regions (ranging from 29 to 57%) and lower in the southern region (ranging from 1 to 35%) [4].

The main goals of the current National Malaria Strategic Plan 2017–2020 of the Mozambican National Malaria Control Programme (NMCP) are to reduce malaria morbidity and inpatient mortality at the national level by at least 40% by 2022 when compared to levels observed in 2015; in order to achieve this goal, targets provide at least 85% coverage of the population with a minimum of one-vector control intervention in every district of the country by 2022 [2]. To reduce the burden of the disease by prevention, among other vector control interventions, the NMCP has proposed vector control intervention by Indoor Residual Spraying (IRS), which is indicated to be primarily used to control the spread of the disease in targeted areas where insecticide resistance (mainly to pyrethroid insecticide on the long-lasting insecticidal bed nets) is reported, in areas where the country is transitioning towards elimination, and in areas with high-transmission intensity to reduce burden [2].

In Mozambique, the implementation of IRS started in 1946 with organochlorines-dichlorodiphenyltrichloroethane (DDT) insecticide, however, only in urban areas of Maputo Province, in the southern region of the country [5, 6]. It was interrupted during the civil war period (1977–1992) and resumed on a small scale in 1994 with pyrethroid insecticides, covering peri-urban and urban areas of Maputo Province [5, 6]. In the early 2000s spraying and coverage were intensified and became regular in almost all provinces in the south region of the country bordering eSwatini and South Africa, with the support of a private initiative called the Lubombo Spatial Development Initiative (LSDI), between the governments of Mozambique, eSwatini, and South Africa [6]. The LSDI implemented an insecticide resistance monitoring programme in southern Mozambique that showed in 1999 that mosquitoes are resistant to pyrethroid insecticides. This resulted in an informed insecticide policy change to the carbamate bendiocarb insecticide for IRS. Bendiocarb insecticide was sprayed in southern Mozambique until 2005, when, due to the high economic costs associated with this insecticide, an operational change was made back to DDT insecticide [5]. Support from LSDI was interrupted in 2011 due to financial constraints [7]. Since 2003, with the advent of the Global Fund to fight AIDS, Tuberculosis, and Malaria (Global Fund) and the President’s Malaria Initiative (PMI) since 2007, the NMCP reintroduced IRS with pyrethroid insecticides in selected districts across the country [6]. Since 2005, the NMCP have extended IRS spraying into the whole country in annual rounds with pyrethroid (where no resistance is reported) and DDT insecticides, until recently, no resistance to DDT insecticide has been detected in the country [1, 6]. Thus, due to the low cost and longer residual decay rates compared to other insecticides, IRS campaigns are heavily dependent on DDT and pyrethroids insecticides in the country [1, 6, 8].

Experience in many African countries has demonstrated the effectiveness of IRS and further work is presented in this topic [9]. To ensure optimal results of the intervention, the latest Global Technical Strategy and Targets for Malaria 2016–2030, ratified in the 68th World Health Assembly, recognized the need for respecting a set of crucial recommendations, including the scheduling of spraying rounds to coincide with the build-up of vector populations just before the onset of the peak transmission season [10].

The results of the assessment of estimated resource needs and impact of the Mozambican National Health Sector Strategic Plan (the guiding policy document for the Ministry of Health of Mozambique [MISAU] for the period of 2014 to 2019) show that the NMCP was considered the costliest of the seven Public Health Programmes within the MISAU, costing nearly US$310 million. This is approximately 20% of the total US$1404 billion estimated for NMCP’s expenditure for the period of 2014 to 2019 [11]. From these estimated needs, 79% of the total were related to medicines and commodities; 10% to programme management (including operational costs, 7%, and training costs, 3%); communications, media, and outreach, 10%; and infrastructure and equipment costs 1% [2, 11].

Funding to implement IRS and other malaria control interventions through the NMCP in Mozambique has for the majority been obtained from international sources, mainly from the Global Fund and PMI, which are estimated to currently be contributing 37% and 39%, respectively, to the total NMCP costs [2]. Support from the Global Fund and PMI guaranteed funds mainly for the purchasing of drugs, insecticide commodities, and equipment, which made up approximately 80% of malaria program costs in the period of 2014–2019 [2, 11, 12]. Other international sources of funds include the United Nations Children’s Fund (UNICEF), WHO, Spain, and the Netherlands [2]. The government budget of Mozambique is another source of funding, estimated to be contributing about 20% of the funds to the NMCP; however, unlike the Global Fund and PMI, it primarily covers mostly NMCP salaries and operational costs (including fuel, training, supervision) at the provincial and district level, in addition to guaranteeing required infrastructure [2, 11].

Until recently, most of government funds came from the Official Development Assistance (ODA), an external financing scheme financed mainly through the World Bank and International Monetary Fund in the form of loan and grants. To illustrate, according to the national Ministry of Economy and Finance (MEF), in 2014 it was estimated that 77% of the general government budget funding was from ODA [13]. However, despite accounting for a significant chunk of this budget, in the last few years ODA disbursements have been restricted by the donors, due to a breach of ODA disbursement practices by the Mozambican government [14, 15]. As a consequence, the ability of the government budget to sustain operationalization costs for scaling up the NMCP’s current malaria intervention for vector control to achieve the stipulated targets by 2022 is uncertain given the particularly challenging undertaking ODA restrictions that may stress available funding. Additionally, the current COVID-19 pandemic has been showing the potential to place an extra burden on health systems’ financial resources worldwide, and especially in countries deeply dependent on external funds; as those external funds that are currently allocated to malaria programmes may be shifted to sustain COVID-19 costs, thus putting even more stress on the scarce availability of financial resources for malaria interventions.

Therefore, given resource constrains due to ODA restriction and the actual context of COVID-19 pandemic, it is imperative to explore options for IRS implementation within the available budget. Accordingly, this study was aimed at analysing the costs of IRS operationalization carried out in Matutuíne and Namaacha, two districts where IRS operationalization was financed by the government budget, following the cost analysis methodology, and the evidence produced in this study intends to provide decision-makers with insight into where they need to pay close attention in future planning in order to operationalize IRS with the existent budget in the actual context of budget restrictions.

## Methods

### Setting of analysis

The study was conducted in Maputo Province in southern Mozambique, in the districts of Matutuíne and Namaacha, two of the eight districts of the Province. Matutuíne borders with Maputo City in the north, the province of KwaZulu-Natal of South Africa in the south, eSwatini in the west, and with Namaacha and Boane districts in the northwest. In the east, the district is limited by the Indian Ocean. Namaacha district is located in the southwest of the province, and borders with Moamba district in the north, Boane district in the east, Matutuíne district in the south, and with eSwatini and South Africa in the west [16, 17]. Both districts are classified as semi-urban and rural, and are divided into 13 and 9 localities, respectively [16, 17]. By 2014, according to the IRS report of 2014 by the Provincial Health Directorate of Maputo Province (SPS), Matutuíne and Namaacha’s projected population in 2014 was 17,501 and 27,597 individuals, with 7479 and 8838 households, respectively [18]. In both districts, malaria transmission occurs throughout the year, but with most episodes occurring from December to April, coinciding with the rainy season. Together, Matutuíne and Namaacha were considered areas with low risk of malaria transmission in relation to other areas of the Maputo Province; however, in both districts, malaria is the leading cause of demand for health care in health units, and the second cause of mortality after HIV/AIDS [18]. In 2014, annual prevalence of confirmed cases was around 5% and 7% in all age groups in Matutuíne and Namaacha, respectively [18]. Matutuíne and Namaacha’s baseline demographic and socio economic characteristics have been described elsewhere [16, 17].

### IRS campaign of 2014 in Matutuíne and Namaacha

In line with Mozambique’s National Malaria Strategic Plan 2017–2022 to deploys IRS in areas targeted for elimination, in both districts, IRS was implemented by the NMCP trough the district health directorates with DDT and deltamethrin. The combination of the two classes of insecticide was related to the different wall surface types, with deltamethrin sprayed on painted walls (due to the visible residues left when spraying) and those located close to food products, and DDT in houses building using (unpainted) local material [18].

The campaign was one round and was initiated on October 20, 2014, and finalized on December 21, 2014, reflecting a total of 45 days of work. All the districts localities were included in the campaign and combined total of 14,496 households (6123 in Matutuíne and 8373 in Namaacha) were sprayed, corresponding to a coverage of 82% and 95% of the targeted households in Matutuíne and Namaacha, respectively [18]. The components of the costs needed to operationalize IRS campaigns were: activities which were paid by the government budget; costs of recruitment, selection, and training; social mobilization, IRS delivery, and supervision. The field staff involved in IRS for these activities included 26 and 41 spray operators in Matutuíne and Namaacha, respectively, and were organized in both districts into teams comprising from the district health directorate: the district director, the medical chief officer, hospital administrator, two supervisors, and one social mobilization technician that informs the community residents about the purpose of the campaign. Additionally, one driver and one warehouse manager were part of the team in each district [18]. On the other hand, the following cost components were paid by the Global Fund budget: insecticide (chemicals and commodities). The sources of financing are detailed in Table 1.

**Table 1:** Cost components and source of financing

Activity	Items	Source of financing
Recruitment and selection of spray operators	Salary	Government budget
Training of spray operators	Stationery material and salary	Government budget
Social mobilization of community	Fuel and salary	Government budget
Supervision	Salary	Government budget
IRS delivery	Insecticides, rental of vehicles, and fuel	Global fund and Government budget

### Cost analysis

#### Identification, measurement, and valuation of costs

A retrospective cost analysis was conducted following an ingredients-based approach (meaning that all resources used in the intervention were identified, measured, and valued) from the provider perspective for a 1-year period, 2014, with costs presented for the IRS operationalization. The year of analysis, 2014, was chosen as operational costs of IRS in both districts were funded by the government budget and were thus susceptible to shocks due to ODA funding restrictions; additionally, 2014 was taken into consideration as it was the last year IRS was implemented before it was resumed in 2018 in these sites due to budget constraints. This informed the choice of districts as the study settings and the year of analysis of the study.

This study adopted the principles of cost analysis [19–21]. The economic costs considered in the study consisted of the financial costs of resources paid by the government budget and other resources provided by donors. All costs were collected and handled in the local currency Meticais (MZN), but for the purpose of publication were converted into United States dollars (US$) using the official exchange rate of the year of the analysis, 2014, provided by the Central Bank of Mozambique (1 US$ corresponds to 30.57 MZN) [22]. Details on the cost data in both settings were gathered from sources including records of expenditure, purchase inventories, official reports, and national and international market prices from WHO-CHOICE [23]. This data was complemented by data on the average population size obtained from the National Census carried out in 2017, as produced by the Mozambican National Institute of Statistic (INE) [24]. Additionally, it was complemented with the number of households sprayed based on data from the reports of the IRS operationalization cycle of 2014 [18]. Details of data sources are shown in Table 2.

**Table 2:** Sources for data collection

Information	Data required	Type of document	Source
Salaries for recruitment and selection, training, social mobilization, and supervision	Staff salary and working hours	Expenditure on salaries in 2014 and 2014 IRS annual financial report	2014 IRS report the SPS
IRS insecticides and commodities and stationery	Prices of resources (unit) and quantities consumed	Expenditure records, purchase invoices	MISAU
Transport (rental and fuel costs)	Total annual fuel and rent costs, and quantities consumed	Expenditure records	2014 IRS report by SPS
Maintenance of vehicle (material, lubricants, fuel, fees, batteries, and spare parts)	Total annual maintenance and rent costs, and quantities consumed	Expenditure records	2014 IRS report by SPS
Utilities (water, telephone, cleaning materials, security services, office supplies)	Total annual costs	Expenditure records	2014 IRS report by SPS
Building	Equivalent price per square meter, interest rate for annualized factor, life years	Local market prices	SPS
Equipment, furniture, and fixtures	Equivalent price per resource, interest rate for annualized factor, life years	Local market prices and standard tables on international costs and prices	Local market and WHO-Choice Analysis publications
Vehicle	Equivalent price per resource, interest rate for annualized factor, life years	Local market prices	Local market
Useful years of life	Number of years of each capital resource	Standard tables on useful years of life of resources	WHO-Choice Analysis publications [23]
Annualized factors	The result of interest rate and useful years of life	Book: Methods for the Economic Evaluation of Health Care Programmes	Drummond et al. [19]
Official exchange rate	Official exchange rate between MZN and US$ in 2014	Official documents from the Central Bank of Mozambique [22]	Central Bank of Mozambique [22]
Interest rate	Official interest rate in 2014	Official documents from the Central Bank of Mozambique [22]	Central Bank of Mozambique [22]
Number of households covered	Number of households covered by IRS operationalization in 2014 in the settings of analysis	2014 IRS annual report	2014 IRS report by SPS
Average size of population per household	Average number of people living in one household in the settings of analysis	National population census of 2007	National Institute of Statistics (2017)

For the analysis, costs were broken down into the following categories:

- *Recurrent costs*, which included (i) personnel costs (salaries of the staff involved in the activities that included recruitment and selection, training, IRS deliver and supervision); (ii) intervention costs, including insecticides (chemical DDT and deltamethrin) and insecticides commodities, such as spray pumps. Other intervention costs included stationery material, transport (rent, maintenance, and fuel costs). (iii) Overhead costs (electricity, water, telephone and cleaning materials).
- *Capital costs*, which included the building, the share of the IRS office space, office space accountability and storeroom, equipment, furniture, and fixtures and vehicles. To estimate the (i) personnel costs, daily wages were multiplied by the number of days worked on IRS operationalization; (ii) the intervention costs of IRS were estimated by multiplying the quantities of units consumed by the price for each unit resource; the total annual cost of transport was divided into two categories: rent of vehicles, and fuel costs. These costs were estimated on the basis of the number of days that the resource was allocated to IRS multiplied by the estimated daily costs of rent or fuel; the maintenance costs were estimated based on the total annual cost of maintenance multiplied by the proportion of days, in a year, that the vehicle was allocated to IRS operationalization; (iii) the overhead costs were estimated based on the total annual costs multiplied by the percentage of staff time devoted to IRS operationalization. To allow for the calculation of opportunity cost and depreciation, the cost of capital resources used by IRS was valued based on the equivalent annual costs approach by annualizing the present value of the resources on the year of analysis over the annualized factor. The resulting annualized costs were extracted from standardized tables [19] and were based on data such as the useful life of the capital resource, following WHO-CHOICE [23]: 40 years for buildings, 5, 7, and 8 years for various furniture and equipment, and 7 years for vehicles. The national interest rate (8%) was given by the Central Bank of Mozambique [22]. The purchase price of the resource in the year of analysis was extracted from the national and international market based on data from the national suppliers and standardized international prices available on WHO-CHOICE [23].

The primary outcomes of the study were calculated and presented as total annual economic and financial costs, economic and financial costs per household sprayed, and economic and financial costs per person protected. All recurrent and capital costs were summated and presented as a total amount across both district settings, and for each district separately. The total economic and financial costs per household sprayed were estimated by dividing the total cost by the number of households sprayed. The total economic and financial costs per person protected were estimated by multiplying the average number of persons per household by the number of households sprayed, with the numerator remaining as the total cost.

### Sensitivity analysis

The last part of the cost analysis calculation included a sensitivity analysis. Hence, an extended “one-way” sensitivity analysis was performed on the key variables to test the uncertainty and assumptions of this study. Vehicles, personnel, and IRS insecticides were tested in a range of ± (above and under) 25% change in all cases was assumed. However, it should be noted that this choice in range variation was arbitrary once the baseline values were taken from the results of the study, and no confidence intervals were calculated to fix the minimum and maximum values on variation. This arbitrary choice of a ± 25% fluctuation range is based on the literature of other studies that reported the analysis of IRS cost. It is assumed that a ± 25% change should be able to identify a more meaningful value change for those variables selected.

## Results

### Total annual economic and financial costs; economic and financial costs per household sprayed; and economic and financial costs per person protected

Tables 3 and 4 present the four identified cost categories and summarize the estimates of the total economic and financial costs, economic and financial costs per household sprayed, and economic and financial costs per person protected. The total annual economic cost for providing IRS operationalization in 2014 was higher in Namaacha (US$128,480.33) as compared to Matutuíne (US$106,222.36). With respect to the financial analysis, there was a total annual financial cost of US$71,781.95 in Matutuíne and US$66,567.70 in Namaacha. Across both settings, the average annual economic cost was US$117,351.34, while the average annual financial cost was US$69,174.83. With regard to the cost per household sprayed, the average annual economic cost per household was estimated at US$16.35, ranging from US$17.35 in Matutuíne to US$15.34 in Namaacha. The average annual financial cost per household sprayed was estimated at US$9.84 (US$11.72 in Matutuíne and US$7.95 in Namaacha). Finally, the economic cost per person protected was US$4.34 and US$3.84 in Matutuíne and in Namaacha, respectively. The financial cost per person protected was slightly higher in Matutuíne (US$2.93) as compared to Namaacha (US$1.99). The average cost was estimated to be US$2.46.

**Table 3:** Economic costs (total annual costs, cost per household (per HH) sprayed, and cost per person (PP) protected)

Categories	Matutuíne				Namaacha				Both districts			
	Total US$	Per HH sprayed US$	Cost PP US$	Total (%)	Total US$	Per HH sprayed US$	Cost PP US$	Total (%)	Total US$	Per HH sprayed US$	Cost PP US$	Total (%)
Intervention	20,628.12	3.37	0.84	19	22,724.40	2.7	0.68	18	21,676.26	3.04	0.76	19
Personnel	29,445.16	4.81	1.2	28	23,849.30	2.8	0.71	19	26,647.23	3.83	0.96	23
Overhead	5,865.18	0.96	0.24	6	6,031.98	0.7	0.18	5	5,948.08	0.84	0.21	5
Capital	50,284.89	8.21	2.05	47	75,876.64	9.1	2.27	59	63,079.77	8.64	2.16	53
Total	106,222.36	17.35	4.34	100	128,480.33	15.3	3.84	100	117,351.34	16.35	4.09	100

**Table 4:** Financial costs (total annual costs, cost per household (per HH) sprayed, and cost per person (PP) protected)

Categories	Matutuíne				Namaacha				Both districts			
	Total US$	Per HH sprayed US$	Cost PP US$	Total (%)	Total US$	Per HH sprayed US$	Cost PP US$	Total (%)	Total US$	Per HH sprayed US$	Cost PP US$	Total (%)
Intervention	20,628.12	3.37	0.84	29	22,724.40	2.71	0.68	34	21,676	3.04	0.76	31
Personnel	29,445.16	4.81	1.20	41	23,849.30	2.85	0.71	36	26,647	3.83	0.96	38
Overhead	5,865.18	0.96	0.24	8	6,030.98	0.72	0.18	9	5,948	0.84	0.21	8
Capital	15,843.49	2.59	0.65	22	13,963.01	1.67	0.42	21	14,903	2.13	0.53	23
Total	71,781.95	11.72	2.93	100	66,567.70	8.0	2.0	100	69,175	9.8	2.46	100

**Fig. 1:**
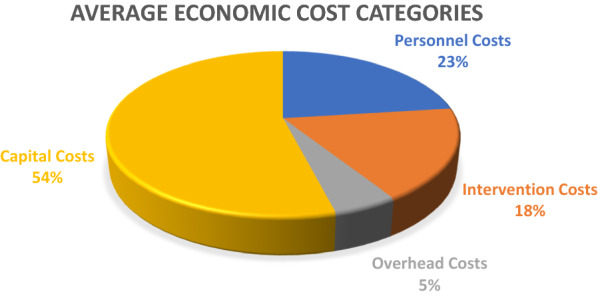
Distribution of the average economic costs categories (%)

**Fig. 2:**
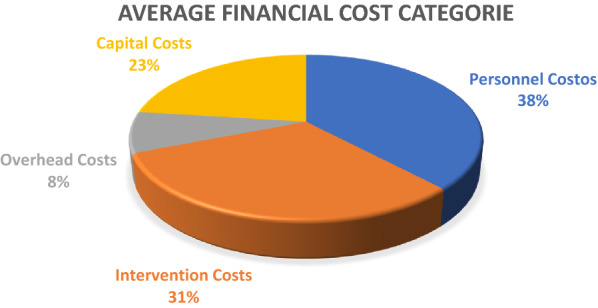
Distribution of the average financial costs categories (%)

**Fig. 3:**
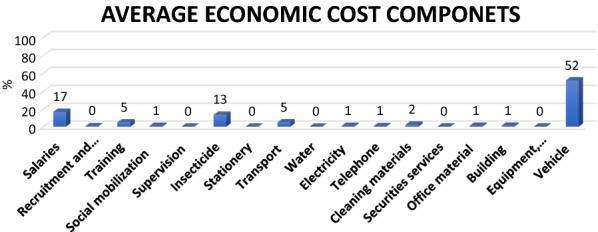
Distribution of the economic cost components

**Fig. 4:**
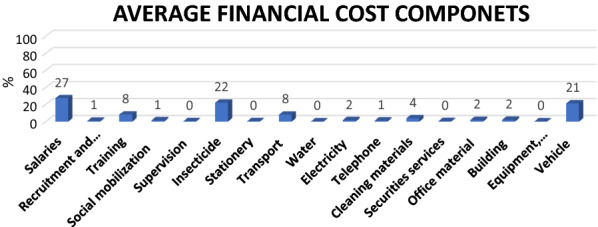
Distribution of the average cost components

Figures 1 and 2 show the distribution of the average cost categories. In the economic analysis, the capital was the most expensive cost category for both settings, accounting for 53% on average. Personnel costs involved in the intervention operationalization were the second highest category in this analysis, comprising on average 23%. Intervention and overhead costs were the lowest costs, sharing on average 19% and 5% respectively of the total cost in the economic analysis. Regarding the financial analysis, personnel costs were the most expensive category at 38%. Intervention costs were the second highest category at 31%. Capital and overhead costs accounted for 23% and 8% of the financial cost. In Figs. 3 and 4, the costs are split into their components. These figures show that the vehicles, salaries, and insecticides were the most expensive items, accounting for 52%, 17%, and 13% of the total economic cost and 21%, 27%, and 22% in the financial analysis.

### Sensitivity analysis

**Fig. 5:**
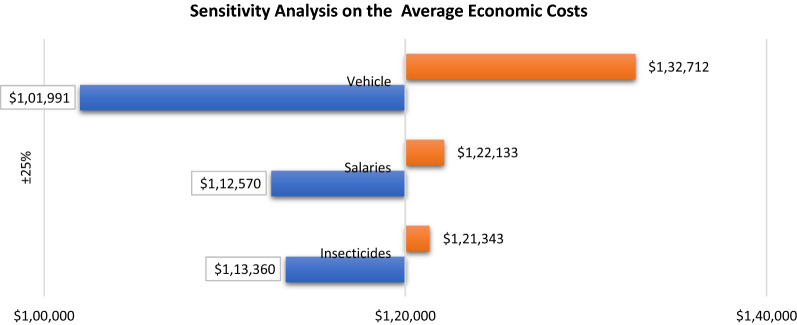
A tornado diagram summarizing the impact of the “one-way” sensitivity analysis on the annual average economic costs

**Fig. 6:**
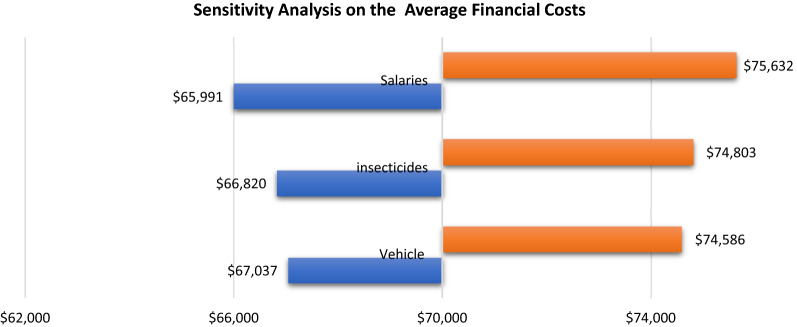
A tornado diagram summarizing the impact of the “one-way” sensitivity analysis on the annual average financial costs

Figures 5 and 6 summarize the findings from the “one-way” sensitivity analysis. The key primary parameters included were the costs of the vehicles, salaries and insecticides. In the economic analysis, the sensitivity analysis on the vehicles had the largest impact on the total average economic cost. To illustrate, there was an approximate change of 14% in the average economic cost when vehicle costs were decreased by 25%. In addition, the average economic cost rose by 11% when the same costs were increased by 25%. Salary and insecticide costs contributed to a change of 4% and 3% respectively on the average economic costs when these costs were deceased by 25%. Furthermore, in the financial analysis, salaries had the most relevant impact. To illustrate, when salaries were changed by 25%, the average financial cost changed by approximately 7%.

## Discussion

Estimates of the total economic cost show that, per year, the IRS operationalization cost is US$117,351.34 on average. In the financial analysis, the average cost totaled US$69,174.83. Furthermore, this study revealed that the main components drivers of the cost of IRS in both settings are vehicles, salaries and insecticides costs, accounting for 52%, 17%, and 13% of the total economic cost and 21%, 27%, and 22% in the financial analysis.

In this current study, the economic cost per person protected was estimated to be US$4.34 in Matutuíne and US$3.84 in Namaacha, and the financial cost per person protected was estimated to be US$2.93 and US$1.99 in Matutuíne and Namaacha, respectively. Although the application of costs analysis received considerable attention in evaluating costs of implementing malaria interventions, it is important to recognize that, to date, few cases of single cost analysis for IRS have been drawn up. In developing countries, most of the few available data are relatively old [25–27]. These studies estimated that the financial and economic costs per person protected ranged between US$0.86 and US$3.48. To illustrate, the study which was done in the highlands of Kenya, found that the financial cost for protecting one person by IRS was US$0.86, while the economic cost was US$0.88 [25]. The study in southern Mozambique found that the economic cost per person protected per year by using IRS in rural areas was US$3.48 and US$2.16 in peri-urban areas, while the financial cost for rural areas was US$3.86 and US$2.41 for peri-urban areas [26]. The study in KwaZulu-Natal, South Africa, found cost per person protected by IRS was estimated in US$1,42 [27]. Several factors could be considered in compering these cost disparities. For example, the lower costs presented in the studies done in Kenya and South Africa compared to the present study, could be due to the insecticides used. Have been reported that in early 2000s within several Africa countries were primarily using DDT due to the low cost and longer residual decay rates compared to other insecticides [8]. However, at this time, in Mozambique, DDT was shifted to a considerable costly insecticide, carbamate bendiocarb, for IRS until 2005 [6, 8]. Thus, this difference on the insecticide used may have influence the high estimates in the current study and in the study in southern Mozambique. Additionally, the analysis of combined interventions in the study done in Kenya that focused on the analysis of a combined malaria intervention of IRS and the distribution of long-lasting insecticidal bed nets, which can generate cost savings [25].

Thus, this may reduce personal costs for each intervention and would plausibly result in lower costs overall. The other factor to consider is how these costs might differ by country-specific characteristics and the conditions of intervention adoption. Heterogeneities in factors such as target region and population size could have impacted transport and logistical costs. On the other hand, assuming the use of 2014 data, the results of the current study should not be interpreted as conservative estimates as recent studies done in Africa and other settings have been also presenting similar results. For example, a cost-effectiveness of a combined intervention of long-lasting insecticide-treated bed nets and IRS spraying in Ethiopia, and found the unit cost of malaria prevention with IRS alone per person protected was US$3.07 [28]. Programmatic analyses done in Mozambique in 2014, show that cost per person protected in was US$ 2.26 [7]. The report that estimated the resources needed to implement the Health Sector Strategic Plan (PESS) for the period of 2014–2019, found that IRS operationalization began the third malaria intervention control with the highest unit costs (US$2) [11].

This study aims to offer an important contribution to the government of Mozambique, by providing evidence of the magnitude of IRS operationalization costs, to shed light on concerns about budget constraints. With the NMCP’s intention of scaling up IRS and other vector control interventions to target at least 85% coverage of the population by 2022, evidence shows major players of IRS financing are external, guaranteeing funds for vehicles and insecticides all corresponding to a share of the total combined average costs of 65% (vehicles, 52% and insecticides, 13%) of the total economic cost and 43% (vehicles, 21% and insecticides, 22%) of the financial analysis.

The cost of vehicles was approximately US$61,440 on average, corresponding to 52% and 21% of the total average economic and financial costs respectively. In this analysis, decreasing vehicle costs by 25% reduced the average economic cost by 13%, from US$117,351.34 to US$101,990.66. On the other hand, in relation to the average financial cost, when vehicle costs decreased by 25%, the average financial cost decreased from US$69,174.83 to approximately US$66,371.71, showing a change of approximately 5%. These results show that vehicle costs do not influence the financial analysis of IRS operationalization much. In fact, one observation of this study is that district health directorates have traditionally operationalized IRS using donated vehicles to reach the targeted households. Instead, the cost of insecticides was approximately US$16,000 on average, corresponding to 13% and 22% of the total average economic and financial costs, respectively, making this the second cost driver in the financial analysis.

As the results show, donor contributions currently guaranteed funds for IRS insecticides, insecticide commodities, and vehicles, meaning that no cash expenditure or financing from the national government budget was used to purchase these items. However, in the context of budget restrictions and high dependence on external resources, it is also important to consider the impact of the COVID-19 pandemic and the consequence on the sustainability of critical public health programmes, as it causes high-level expenditure and has dominated priorities in the political agendas of governments, particularly in the global health sector. This has led to prioritization of resource allocation and making governments have to deal with challenges to poll resources that are constrained to respond to the pandemic, which can lead to malaria programmes being left without financial resources as has already been reported, and so increasing scepticism that investment in malaria control can be neglected [30–32]. Thus, the government of Mozambique should be aware of the impact of COVID-19, as those external funds that are allocated to malaria programmes may be shifted to sustain COVID-19 costs.

Personnel salaries which were allocated from the government budget to the district health directorate in the year of analysis were the largest cost driver in the financial analysis, sharing 27% of the total average financial costs. In this analysis, the cost components that comprised personnel salaries were as follows: recruitment and selection (US$521), training (US$6,000), social mobilization (US$970), supervision (US$213), and IRS delivery by spray operators (US$19,300). Meaning that, the personnel salaries cost was mostly driven by costs of spray operators, followed by the cost of training. On the other hand, the sensitivity analysis shows that after reducing this cost by 25%, the average economic cost decreased by approximately 4%, from US$117,351.34 to US$112,461.68, and the average financial cost decreased by approximately 7%, from US$69,174.83 to US$64,285.17. Considering personnel salaries as the main cost driver of the financial analysis and spray operators’ costs as the driver of personnel salary, it is important also to note that largely due to the reduction in ODA funding, it has been documented that recurrent spending in the health sector has been reduced which has been inconsistent and declining since 2014 [2]. Thus, a suggestion to reduce costs could be if for example spray operators are effectively utilized, some of the operational cost items can be eliminated from the cost of operation for IRS implementation, hence lowering the financial resources needed. In Mozambique, new spray operators are hired for every IRS campaign on a seasonal basis (in accordance with the IRS length); this included costs of recruitment and selection, as well as malaria surveillance and entomological costs. One suggestion is to keep the same spray operators at least while the same insecticides are used in the regions, assuming that no training for the introduction of new insecticides would be needed; this would thus reduce the costs of recruitment, selection, and training that together made up 9% of the total average financial costs. Further cost-reduction strategies involve options such as the use of Community Health Workers as spray operators, ensuring that IRS operationalization is incorporated into their day-to-day activities. This can eliminate or greatly minimize the cost of personnel salaries as the cost of recruitment, selection, and training can be avoided.

Understanding how IRS timing affects the effectiveness of the intervention can further increase impact and would help optimize the deployment of this intervention under budget constraints. Based on the results, and thus, for optimizing IRS operationalization within the available budget, the government of Mozambique should consider continuing targeted spraying, taking into consideration the correct timing for IRS operationalization. It was reported that during the 2014 IRS cycle period in Matutuíne and Namaacha, the spraying round should have been initiated in October during the wet season, and so prior to peak vector abundance; however, the cycle was initiated in December, which corresponds to the peak in vector abundance [18]. Evidence has demonstrated the efficacy of IRS when considering the correct sequential timing and seasonality of the disease [10]. The evidence showed that earlier spraying is related to more effective results in reducing malaria prevalence [25, 32–34].

## Study limitations

The collection of cost data was the most likely source of information bias in this study. For instance, some information and data regarding intervention costs (specifically material and supplies) were not found in the setting of analysis, thus it was exported from the SPS in Maputo Province. Therefore, data was assumed to be correct and the information was used for both settings of analysis. However, our estimates may be conservative as this information may, therefore, be subject to biases that may have influenced the study estimates. To minimize this bias, source documentation for key data, namely accounting spreadsheets, was reviewed for quality.

The other limitation that this study faced was that it takes into consideration settings that had the government budget as the main source of financing for IRS operationalization activities. The fact that the source of funding has been restricted by ODA may influence the reduction of resources to spend during IRS operationalization. In some instances, the SPS reported constant cuts on the planned activities for the 2014 IRS operationalization due to lack of budget. However, additional factors affecting costs need also be considered in extrapolating these results to other settings.

Additionally, the lack of cost analysis of IRS studies in Africa setting included Mozambique constituted also a limitation for the present study. As such, it was difficult to compare the findings of the study to other studies. This shows a gap in the literature regarding full and single cost analysis of IRS studies to inform decision makers regarding the costs of this important intervention to control malaria. This highlights the urgent need for research to address this shortfall. Thus, based on this evidence, further works in the line of this study are suggested.

## Conclusions

A cost analysis of the 2014 cycle of IRS operationalization (from October 20, 2014, to December 21, 2014) was conducted in two Mozambican districts. Among all costs, vehicles, salaries and insecticides, were the main drivers. Altogether, the current cost analysis provides an impetus for the consideration of targeted IRS operationalization within the available governmental budget, in accordance with IRS rounds being correctly timed to coincide with the build-up of vector populations. Additionally, to sustain IRS operationalization, the focus should be on the efficient use of spray operators. This study used data from 2014. However, results from the study may not be considerable conservative as they are in line with the recent estimates. Nevertheless, further cost analysis studies to inform the government regarding the impact of ODA restrictions on the sustainability of IRS operationalization using more recent data are suggested.

## Data Availability

The supporting data for this article will be shared by the corresponding author upon reasonable request.

## Abbreviations

DDT: Dichloro-diphenyl-trichloroethane
Global Fund: Global fund to fight AIDS, Tuberculosis, and Malaria
INE: Instituto Nacional de Estatística (National Institute of Statistics, in Mozambique)
INS: Instituto Nacional de Saúde de Moçambique (National Health Institute in Mozambique)
IRS: Indoor residual spraying
LSDI: Lubombo Spatial Development Initiative
MEF: Ministério da Economia e Finanças de Moçambique (Ministry of Economy and Finance in Mozambique)
MISAU: Ministério da Saúde de Moçambique (Ministry of Health of Mozambique)
MZN: Meticais, Metical (the official currency of Mozambique)
NMCP: National Malaria Control Programme
ODA: Official Development Assistance
PMI: President’s Malaria Initiative
SPS: Serviço Provincial de Saúde de Maputo Provincia (Provincial Health Service of Maputo Province)
UNICEF: United Nations Children’s Fund
US$: United States dollars
WHO: World Health Organization
